# The Presidency of the Congress

**Published:** 1886-04

**Authors:** 


THE PRESIDENCY OF THE CONGRESS, MADE VACANT BY THE DEATH OF DR. FLINT.

The *Mississippi Valley Medical Monthly*, in nominating "Our Own" N. S. Davis for this high and responsible position, says:

"Dr. Davis has an international reputation not inferior to that of any American author, teacher, or practitioner. He is the father of the American Medical Association, and, like Dr. Flint, has been a devoted friend throughout its entire history. His busy pen has made every part of the globe, where medicine has a scientific basis, familiar with his teachings. His last production, a systematic treatise on the 'Principles and

Practice of Medicine,' has no superior from the American press.

"If, then, we would add to the laurels of American Medicine,—secure a successful Congress in the face of a factious opposition, and maintain our national organization by continuing the unity of the great mass of our profession, Dr. Davis will be the proper man for the vacancy. It would appear quite a fitting tribute to one who has been so devoted to the cause of organized medicine to be privileged to round up a lifetime of devotion to the profession with the highest honors within the gift of the entire profession."

We heartily endorse every word of the above, and join our able cotemporary in urging the fitness of Dr. Davis for the succession, above, perhaps, any man in America.

